# Does Quantitative Research in Child Maltreatment Tell the Whole Story? The Need for Mixed-Methods Approaches to Explore the Effects of Maltreatment in Infancy

**DOI:** 10.1155/2016/1869673

**Published:** 2016-08-11

**Authors:** Samuel Glass, Ruchika Gajwani, Fiona Turner-Halliday

**Affiliations:** Institute of Health & Wellbeing, University of Glasgow, Academic Unit of Mental Health & Wellbeing, Caledonia House, Royal Hospital for Sick Children, Yorkhill, Glasgow G3 8SJ, UK

## Abstract

*Background and Aims.* Research on child maltreatment has largely overlooked the under-five age group and focuses primarily on quantitative measurement. This mixed-methods study of maltreated children (*N* = 92) entering care (age 6–60 months) combines a quantitative focus on the associations between care journey characteristics and mental health outcomes with a qualitative exploration of maltreatment in four different families.* Methods.* Care journey data was obtained from social care records; mental health and attachment assessments were carried out following entry to care; qualitative data comprised semistructured interviews with professionals, foster carers, and parents.* Results.* Significant associations were found between suspected sexual abuse and increased DAI inhibited attachment symptoms (*p* = 0.001) and between reported domestic violence and decreased DAI inhibited (*p* = 0.016) and disinhibited (*p* = 0.004) attachment symptoms. Qualitative results: two themes demonstrate the complexity of assessing maltreatment: (1) overlapping maltreatment factors occur in most cases and (2) maltreatment effects may be particularly challenging to isolate*. Conclusions.* Qualitative exploration has underscored the complexity of assessing maltreatment, indicating why expected associations were not found in this study and posing questions for the quantitative measurement of maltreatment in general. We therefore suggest a new categorisation of maltreatment and call for the complimentary research lenses of further mixed-methods approaches.

## 1. Background

As of 2013, the number of children in care in the UK was 92 000 and this figure continues to rise [1]. The reasons for children entering care are varied but evidence suggests that the overwhelming majority have suffered maltreatment [2–5]. Factors such as poor physical environment, poverty, social/cultural norms, and existing parental/child psychopathology interact to shape a child's care and maltreatment experiences [6]. Research suggests that children in care suffer from poor overall health [7, 8]. Prevalence of chronic medical problems [7], developmental problems [7, 9], and physical and learning disabilities/difficulties [3, 10] have been shown to be high and children in care are more likely to have been small for gestational age or premature [7].

Maltreatment has been shown to lead to both lower levels of secure attachment and poor mental health outcomes [4, 11], with research suggesting negative mental health outcomes to be present in 45–60% of all children in foster care [2, 4, 12]. Several factors in the care experiences of maltreated children have been linked to increased likelihood of negative mental health outcomes and poor attachment, including specific types of maltreatment such as sexual or physical abuse [13], multiple types of maltreatment [14], and placement instability [4, 15]. Recently the concept of Maltreated Associated Psychiatric Problems (MAPP) was presented as a multidimensional way of thinking about the overlap of effects that maltreated children present with, highlighting the complexity of maltreatment effects on young children [16]. Work by the Centers for Disease, Control and Prevention in the US has found that adverse childhood experiences (referred to as “ACEs”) are strongly related to the development and prevalence of risk factors for disease, health, and social wellbeing [17]. These problems can continue throughout the lifespan and some have been found to lead to suicide [18], criminality [19], and substance misuse [18]. However, factors that may promote resilience amongst maltreated children are currently being unearthed and it is known that recovery from the effects of early maltreatment can be rapid and remarkable if safe, nurturing care is achieved early enough, ideally in the first year of life [3, 4, 20].

The majority of studies use quantitative methods to look for associations between various aspects of care journeys and mental health and focus on children past the age of infancy. Therefore there are gaps in our understanding of the ways in which maltreatment and care experiences may affect younger children. Furthermore, there may be limitations to using a primarily quantitative approach to study maltreatment; as discussion regarding the overlapping nature of MAPP reminds us, complex processes can be better studied using mixed-methods approaches that also provide a complimentary qualitative approach to provide depth and context to the variables being studied. Pertinent to the findings later presented in this study, a mixed-methods approach often uses the qualitative component to examine, in greater depth, findings generated by a preliminary quantitative approach that may be surprising or inconsistent [21].

Given the gaps in quantitative research looking at impact of maltreatment and care experiences on infants and the relevance of a mixed-methods approach to (a) researching maltreatment as a complex process and (b) examining the possible reasons for surprising quantitative findings, this paper therefore presents the findings of a mixed-methods study of an infant population who have entered care due to maltreatment. Firstly, we profile the care journeys of a sample of children entering care in Glasgow between 6 December 2011 and 23 October 2013. Secondly, we aimed to investigate any associations between characteristics of these care journeys (i.e., specific types of maltreatment, number of types of maltreatment, age at entry to care, and number of carers) with mental health outcomes and attachment. Thirdly, we explore qualitative data pertaining to four families from this cohort in order to illuminate key issues around defining and assessing cases of maltreatment in practice that may impact on quantitative findings.

## 2. Methods

Children were recruited through the Best Services Trial (BeST^?^) between 6 December 2011 and 23 October 2013. BeST^?^ is an exploratory randomised controlled trial (RCT) comparing the New Orleans Intervention Model (NIM: a new multidisciplinary intervention for maltreated preschool children entering care) with existing but enhanced social work based services in Glasgow. Further details of BeST^?^ can be found elsewhere [22].

For quantitative data collection, 92 children were recruited for whom access to care journey data was available. For the qualitative data, four cases were purposively selected: two from NIM and two from existing services that were looking likely to have opposing outcomes in terms of whether the child/ren would return home or not. All four cases were nearing the end of the services' three-month assessment phase at the time of data collection [22] and involved seven children in total. Participants in the case studies included the key people surrounding each family; the parent/s, foster carer/s, area team social worker, and key professionals from the service that was assessing the family in order to make a recommendation about whether the child should return home or not.

### 2.1. Inclusion Criteria

All families with a child aged between six months and five years who entered care in the Glasgow City Council catchment area for reasons associated with maltreatment were invited to take part in BeST^?^.

### 2.2. Exclusion Criteria

Children were excluded from the study if they had a profound learning disability (as assessment outcome measures would not be appropriate) or their primary caregiver was unavailable to take part in the intervention (such as long-term imprisonment, death, or being uncontactable by services or research team for three months or more).

### 2.3. Quantitative Measures

Data concerning the child's care journeys was obtained retrospectively from CareFirst, a social care case management system used throughout the UK by most local authorities but mainly by social work practitioners. Three research assessments were performed over the course of BeST^?^. The assessments included the Infant-Toddler Social and Emotional Assessment (ITSEA) [23–26] to assess mental health and the Disturbances of Attachment Interview (DAI) [27, 28] to assess attachment. Only the baseline assessments which were with foster carers, completed around three months after entry to the study and prior to randomisation to either arm of the BeST^?^study, were used for the purposes of this study.

### 2.4. Qualitative Measures

Eighteen semistructured interviews across the four families were carried out with the aim of exploring perceptions about the different facets of the case, for example, factors surrounding the maltreatment, expected trajectory of the case, interactions between services, and parent engagement in the process. Interviews typically took between 60 and 90 minutes.

### 2.5. Statistical Analysis

Associations between care journey characteristics (type of maltreatment, number of types of maltreatment (≤1 or >1), number of carers (1 or >1), and age at entry to care (≤3 yrs or >3 yrs)) and mental health outcomes were assessed using chi square (*χ* ^2^) tests. DAI inhibited and disinhibited scores were analysed using Mann-Whitney *U* tests for associations with the same care journey characteristics. *t*-tests were used to compare mean DAI scores of this sample to a normalised sample. *p* values <0.05 were considered significant.

### 2.6. Qualitative Analysis

All interviews were audio-recorded and transcribed verbatim. The data was subject to a thematic analysis [29] where recurring patterns were interpreted in the data and organised into themes that captured the essence of different facets of maltreatment experience. For the purpose of this paper, we present two key themes that were found in relation to defining and assessing maltreatment in practice that may impact on the generation of quantitative findings.

## 3. Results

### 3.1. Quantitative

The characteristics of 92 children and their care journeys have been profiled. Due to a large amount of missing data for this sample, there is some variation in the number of children on which each characteristic is reported. The sample (*N* = 92) consisted of 55.4% (51) males and 44.6% (41) females. Mean age at entry to the study was 31.2 months (SD = 16.0). In regard to ethnicity, 85.9% (79) were white British. Of the 87 children on whom birth data was available, 9.2% (8) were born prematurely (born ≥4 weeks before birth due date).

**Figure 1:**
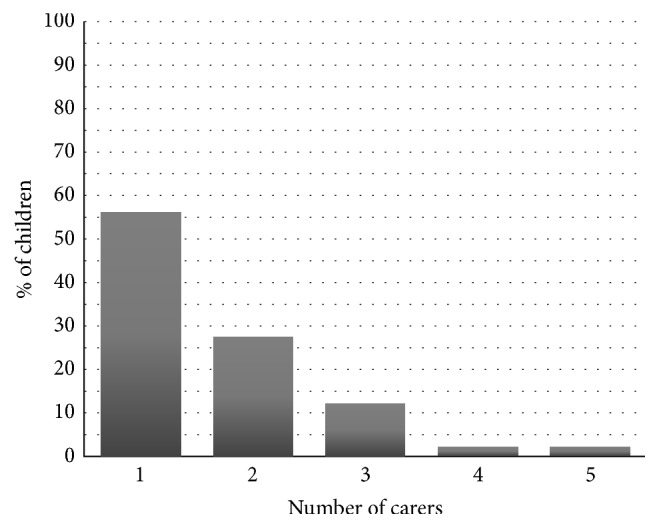
Number of carers per child (updated to 1 December 2013) (*n* = 91).

In this sample (*N* = 92), mean age at entry to first episode of care was 27.8 months (SD = 17.1). As of 1 December 2013, 82.6% (76) of 92 children had entered 1 episode of care and therefore the episode which led to their referral to the trial was their first. 16.3% (15) of the children had entered 2 episodes of care and 1.1% (1) had entered 4. Respite episodes are when a child comes into care on a planned short term basis to give birth parents a break and to prevent the child from entering foster care. Prior to the episode of care which led to referral to the trial, 29.3% (27) of 92 children had experienced respite care. Of this subsample (*n* = 27) who had entered respite episodes, the median of these was 3, with 5 children having over 20 respite episodes. As of 1 December 2013, 44.0% (40) of 91 children had >1 carer (Figure 1). The mean number of carers for all 92 children was 1.7 (SD = 0.9). In their first episode of care, 93.4% (85) of 91 children were placed in foster care and 6.6% (6) were placed in kinship care. This number is not necessarily representative of all children in care as our study only included children brought into an episode of foster care.

The mean age of the mothers (*n* = 77) at the time of the birth of the child involved in the study was 24.9 years (SD = 5.9) with an age range of 15 to 43 years. 10.3% (8) of 78 mothers were reported to have been accommodated at some point as a child. Parental mental health problems were reported in 26.1% (24) and parental learning difficulties in 10.9% (10) of 92 children. Over a third (36.3% (33)) of 91 mothers were with the child's father when they entered the study.

**Figure 2:**
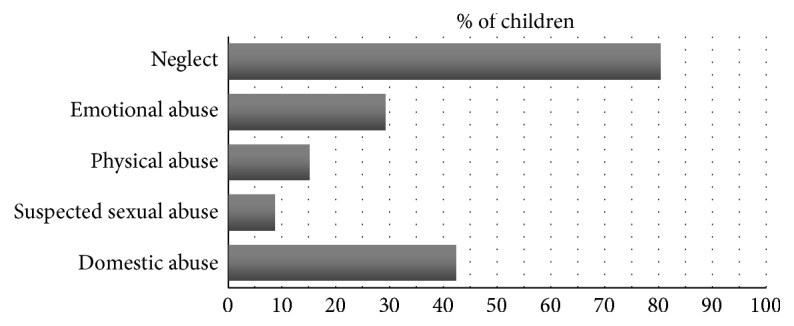
Type of maltreatment reported (*N* = 92).

In this sample (*N* = 92), neglect was the most common reason for initial entry to care (Table 1), reported in 31.9% (29) of 92 children. Neglect was also the most commonly reported form of maltreatment (Figure 2), reported in 80.4% (74) of 92 children. Over half (56.5% (52)) of 92 children reported more than one type of maltreatment with 27.2% (25) reporting three or more. Parental substance abuse was also reported in 53.3% (49) of 92 children (alcohol 13.0%, drugs 26.1%, and alcohol and drugs 14.1%). In 28.3% (26) of 92 children, their physical health problems were implicated in their need for accommodation for at least one episode of care.

65.2% (60) of the 92 children had Infant-Toddler Socio-Emotional Assessments completed. All were aged between 12 and 48 months at assessment. Over half (56.7% (34)) (Table 2) of this subsample exhibited a domain of concern and around a third of these children (32.4% (11)) exhibited more than one domain of concern. The 77 children on whom Disturbances of Attachment Interviews (DAIs) were completed also demonstrated significantly elevated presence of inhibited (*t*(108) = 4.53, *p* < 0.0001) and disinhibited (*t*(108) = 5.36, *p* < 0.0001) attachment behaviours compared to a normative sample of children living at home (Table 3) [28].

No significant associations were found between care journey characteristics (type of maltreatment, number of types of maltreatment (≤1 or >1), number of carers (1 or >1), and age at entry to care (≤3 yrs or >3 yrs)) and prevalence of mental health problems measured by the ITSEA.

Suspected sexual abuse was associated with increased DAI inhibited scores in this sample (*U* = 78.00, *p* = 0.001). No further significant associations were found between care journey characteristics (type of maltreatment, number of types of maltreatment (≤1 or >1), number of carers (1 or >1), and age at entry to care (≤3 yrs or >3 yrs)) and increased symptoms of attachment disturbance (DAI inhibited or disinhibited scores). However, a significant association was found between domestic abuse and both lower DAI inhibited (*U* = 493.5, *p* = 0.016) and DAI disinhibited (*U* = 446.5, *p* = 0.004) scores.

### 3.2. Qualitative

We investigated maltreatment contexts qualitatively with the aim of gaining insight into how the nature of maltreatment contexts may impact on quantitative measurement.

Two main themes were found in relation to defining and assessing maltreatment in practice, which we suggest may have implications for the measurement of maltreatment in quantitative research.

#### 3.2.1. Overlapping, Multiple, and Emergent Maltreatment Factors 

The case studies suggest that a specific maltreatment type is cited when a child comes into care; however, there are often several more specific factors that interrelate:

*The main issue [reason cited for removal] was basically longstanding neglect; really bad neglect, chronic neglect. And there was domestic violence, there was alcohol misuse, there were gambling issues. There were lots of other issues but overall it was ultimately chronic neglect. (Case study 2; area team social worker) *

The identification of multiple overlapping maltreatment factors was described as routine in cases of maltreatment; however, in case study 1, the problems were primarily reported as severe physical neglect in the first instance. At the start of the assessment period, a contrast between the emotional and physical care of the children was conveyed:

*I think in this particular case mum is very good with love, affection, interaction, her contact, there is clearly an attachment there…but she has struggled with practical parenting; the meal times, the bed times, cleaning the house, the bills, getting them to and from health appointments, to nursery. (Case study 1; area team social worker) *

In terms of intervention, maltreatment contexts like case study 1 where emotional care already exists were felt to be more straightforward in their trajectory but unusual in that deficits in emotional care were felt to be more commonly entangled with most forms of maltreatment:

*People can learn to hoover a carpet but it is quite difficult to teach people how to love or be empathetic and that's what's often missing, but in this case it is not missing…they [the kids] are absolutely delighted to see her when they turn up and very, very attached to her…a nice straightforward case, lovely woman, lovely kids, and that's unusual. (Case study 1; service social worker) *

Although the mother in case study 1 made initial progress in practical aspects of parenting and reunification looked likely, during the assessment period she started to show signs of relapse. At this point the case had started to look more complex and identification of emotional and psychological issues in the mother appeared to prompt a more general discussion about whether such severe physical neglect could occur without the relationship between parent and child, and emotional care of the child, being implicated:

*If they [children] have not had that quality of life and care…how does a child know that is a safe person that can meet their needs day in and day out? (Case study 1; area team social worker)*

There appears to be an emerging picture of complexity (through assessment) in cases where the reasons for maltreatment are nonspecific and harder to identify. Case study 3—another case involving both physical and emotional neglect—was proving challenging in terms of defining the underlying causes. Discussion in some of the interviews around “parental choice versus parental ability” suggests that the reasons behind a general inadequacy in parenting are less easy to define than specific problems, for example, addiction, that are already known to be strong determinants of child maltreatment:

*There is no alcohol or drug issue or anything like that which would make it really really clear…this is about parenting and neglect, which is much more difficult to kind of unpick the reasons why and it is much more difficult to do anything about…It is almost like we have a family who we clearly know have difficulties and, you know, it is not by choice; it is about their learning and stuff like that which is very different from somebody making choices to prioritise drugs and chaos over the needs of their children or putting their children at risk. (Case study 3; area team social worker) *

#### 3.2.2. Attributing Causation to Maltreatment 

Another main theme emerges from the case studies centres on the difficulty of differentiating the effects of maltreatment from other causes. In two of the case studies, there was significant complexity in attempting to separate MAPP from other health-related causes of the children's problems. In addition to severe neglect, professionals in case study 3 were exploring the possibility of the child having a neurodevelopmental problem. Similarly, in case study 4, a known physical health condition had complicated the assessment of bruising.

*He was just left along in his room for long periods of time in the dark…no stimulation, no interaction, so his brain development will already be behind what it should be; his physical needs but also his emotional interactions…The medical team were saying we think there might be something there, but we cannot assess him for that [Autistic Spectrum Disorder] because he has got such chronic neglect that it could all be because of that…my own view is that it is a combination of both - I do think there is something underlying but the neglect absolutely compounded it. (Case study 3; area team social worker) *

*They [child's physical health problems] do have an impact on some of the issues which could be confusing and difficult to tease apart from the child protection point of view; for example, she came into care because she had a bruise…now this wee girl does fall over. (Case study 4; service professional)*

On the other side of the coin, it was also felt that other health conditions could mask maltreatment indicators. Alongside this perspective was a perceived value in multidisciplinary assessments of maltreatment involving health and social models:

*When you do have a case like that with a child who has got additional needs things can be masked, like her development…then it indicates that potentially that [bruising] could be part of her condition…whereas now we have got both sides of it [from a health and social work assessment] and it [maltreatment] is not masked by the health condition, it is actually about the interaction [between the child and her parents]. (Case study 4; area team social worker)*

In both case studies, the parents had focused on the health and neurodevelopmental issues as the cause of the children's problems, which was hampering the work of the services in prompting the parents to take ownership of the maltreatment. Interestingly, in both cases, it was the removal of neglect (i.e., the children placed in foster care) and the subsequent development of the children in a short space of time that had seen the assessment service place more weight on maltreatment than on the other health issues as the main cause of the children's problems:

*He came in as a little boy, four years of age, still in nappies and couldn't speak…he couldn't even walk hardly…his body hadn't been used to doing anything other than sitting in his bedroom…he has changed so markedly, I do not even know where to start… he is not the little boy that came into the house six months ago. (Case study 3; foster carer) *

## 4. Discussion

Firstly this paper looked at care journeys. We found that whilst the majority of children in our sample had one care episode, a proportion of the sample experienced considerable instability through respite care episodes. This replicates the results of previous research [5, 30, 31].

Negative mental health outcomes were present in over half of children with ITSEAs completed. One of the most comprehensive studies into mental health of looked-after children in Scotland found 45% of children to have a mental health disorder, around four to six times higher than in the general population of children [12]. In the children with complete DAIs, high rates of inhibited and disinhibited attachment behaviours were also found. This concurs with previous research which has found rates of reactive attachment disorder to be higher in children in care relative to their peers [2]. However, these previous studies on mental health outcomes and attachment excluded children under five years old which this study included. Findings of high prevalence of psychopathology in children under five years old emphasise the need for early intervention in the lives of maltreated children in care.

Although we found a high proportion of our sample had psychopathology, no associations were found between care journey characteristics and mental health outcomes. It is worth remembering, however, that all children in this sample were very young and these findings may not represent their final outcomes. Whilst our study found no significant associations between type of maltreatment and increased mental health disorder prevalence, previous research suggests that physical or sexual abuse may lead to increased mental health need [13].

Assessing different types of maltreatment separately for associations proved challenging in this study as over half of children in this sample experienced more than one type of maltreatment, a finding replicated across studies [32]. Indeed the qualitative data suggests that maltreatment types overlap far more often than not, even though a single type may be cited in the child's record when removed from parental care. The identification of multiple maltreatment types is often emergent as additional factors are subsequently unearthed during assessment, highlighting the possible usefulness of multidisciplinary methods of assessment involving both health and social models. It is clear that emotional factors, in particular, may take time to unearth, as do the reasons for maltreatment in cases where abuse and/or neglect do not appear to be the result of commonly known risk factors. Furthermore, attempting to differentiate between the effects of maltreatment and other health-related problems poses both challenge and debate amongst assessment services. Overall, the experience of those working in the maltreatment field is that combined maltreatment factors, and thus an inherent complexity, are the norm, posing questions about the usefulness of maltreatment research that is based on a single preassessment snapshot in time and that may attempt to artificially separate “real-life” cases of maltreatment into overly simplistic variables. This may provide insight into the lack of associations found in the quantitative component of this study and underscores the usefulness of mixed-methods approaches in research that can give explanatory power to unexpected research findings.

Although associations between care journey characteristics and mental health outcomes were not found, associations with attachment were. A significant association was found between suspected sexual abuse and attachment inhibition. Previous research has shown an association between sexual abuse and increased symptoms of reactive attachment disorder (RAD), though not specifically on the inhibited subscale [33]. It is possible that “grossly pathogenic care” [33] such as that experienced by sexually abused children is causal in attachment disorder development. Our study also found a significant association between domestic violence and lower DAI inhibited and disinhibited scores, suggesting that children who are exposed to domestic violence are less likely to exhibit symptoms of attachment disorders in comparison to other maltreated children.

The associations between attachment and sexual abuse and domestic violence, along with the qualitative findings in this study, may have drawn attention to the possibility of a new classification of maltreatment for research purposes based on complexity of maltreatment rather than type of maltreatment. It is possible that maltreated children who report sexual abuse have more complex maltreatment experiences overall and therefore display greater symptoms of attachment disorder. Conversely, children who report domestic violence may have less complex maltreatment experiences and therefore display less symptoms of attachment disorder compared to other maltreated children. This would suggest that future study should explore the complexity of a child's maltreatment experience and assess associations between this complexity and mental health outcomes.

A major limitation of this study was extensive missing data for both the care journeys and mental health outcomes. Therefore what could be reported on for care journeys was influenced by completeness of the data. Furthermore, frequent underreporting of maltreatment may have influenced analysis. There were particular difficulties with unclear data recorded in CareFirst forms. As in many studies in this field, small sample size in the secondary analysis of mental health outcomes and attachment disorders is a major limitation of this study. Additionally, this study analysis is part of a larger trial (BeST^?^) which is continuing to collect baseline data, limiting confirmatory analysis on care journeys and their relation with mental health outcomes. In order to look at maltreatment, care journeys, and mental health problems of children in care in a meaningful manner, greater collaboration between social and health services is required. Routine data collection should be improved, ensuring accurate recording of relevant information. Additionally, medical data should be sought for future study given suggestions in the qualitative data that health conditions can complicate the relationship between maltreatment and outcomes.

Perhaps it is time to reconsider the way in which we approach the study of maltreatment and generate an informative debate about whether categorising it into maltreatment types is indeed useful in looking for associations with mental health outcomes. The findings of this study highlight that complexity and overlap in maltreatment factors is very much the rule rather than the exception, suggesting that one possible alternative that may warrant further exploration is a classification system for research purposes that separates maltreatment by complexity rather than neglect or abuse type. To inform this dialogue, further qualitative research is also warranted, providing depth to the understanding of the complexity of maltreatment and explanations of the associations found, or not found, in quantitative datasets. This study has shown that complimentary research lenses may be particularly useful to the study of child maltreatment; the value of mixed-methods and longitudinal approaches should not be underestimated in this endeavour. The ongoing BeST^?^ trial [22] will provide an opportunity for follow-up study in this field.

## Figures and Tables

**Table 1:** Main reason reported for accommodation at first episode of care (*n* = 91).

Main reason for first accommodation	Frequency (%)
Neglect	29 (31.9%)
Physical abuse	12 (13.2%)
Emotional abuse	2 (2.2%)
Parent mental health issues	3 (3.3%)
Substance abuse	16 (17.6%)
Parenting issues	13 (14.3%)
Child physical health	1 (1.1%)
Domestic violence	10 (11.0%)
Risk to siblings	3 (3.3%)
Primary caregiver unavailable	2 (2.2%)

*Total*	*91*

**Table 2:** Children with domain of concern on ITSEA (*n* = 60).

ITSEA: domain of concern	Yes (%)
Any	34 (56.7%)
Externalising	17 (28.3%)
Internalising	8 (13.3%)
Dysregulation	3 (5.0%)
Competence	18 (30.0%)

**Table 3:** Mean scores of DAI (*n* = 77) compared to normative sample^*∗*^ [28].

DAI behaviour	Current sample mean (SD)	Home-reared sample mean (SD) [29]
Inhibited (DAIs 1–5)	1.97 (2.13)	0.27 (0.45)
Disinhibited (DAIs 1, 6–8)	2.79 (2.48)	0.42 (0.79)

^*∗*^Data from [28].
